# Towards a scalable approach to assess speech organization across the psychosis-spectrum -online assessment in conjunction with automated transcription and extraction of speech measures

**DOI:** 10.1038/s41398-024-02851-w

**Published:** 2024-03-21

**Authors:** Julianna Olah, Nicholas Cummins, Maite Arribas, Toni Gibbs-Dean, Elena Molina, Divina Sethi, Matthew J. Kempton, Sarah Morgan, Tom Spencer, Kelly Diederen

**Affiliations:** 1 Department of Psychosis Studies, Institute of Psychiatry, Psychology & Neuroscience, King’s College London, London, UK; 2 Institute of Psychiatry, Psychology and Neuroscience, Department of Biostatistics & Health Informatics, King’s College London, London, UK; 3 Behavioural and Clinical Neuroscience Institute, Department of Psychiatry, University of Cambridge, Cambridge, United Kingdom

**Keywords:** Predictive markers, Diagnostic markers, Schizophrenia

## Abstract

Automatically extracted measures of speech constitute a promising marker of psychosis as disorganized speech is associated with psychotic symptoms and predictive of psychosis-onset. The potential of speech markers is, however, hampered by (i) lengthy assessments in laboratory settings and (ii) manual transcriptions. We investigated whether a short, scalable data collection (online) and processing (automated transcription) procedure would provide data of sufficient quality to extract previously validated speech measures. To evaluate the fit of our approach for purpose, we assessed speech in relation to psychotic-like experiences in the general population. Participants completed an 8-minute-long speech task online. Sample 1 included measures of psychometric schizotypy and delusional ideation (*N* = 446). Sample 2 included a low and high psychometric schizotypy group (*N* = 144). Recordings were transcribed both automatically and manually, and connectivity, semantic, and syntactic speech measures were extracted for both types of transcripts. 73%/86% participants in sample 1/2 completed the experiment. Nineteen out of 25 speech measures were strongly (*r* > 0.7) and significantly correlated between automated and manual transcripts in both samples. Amongst the 14 connectivity measures, 11 showed a significant relationship with delusional ideation. For the semantic and syntactic measures, On Topic score and the Frequency of personal pronouns were negatively correlated with both schizotypy and delusional ideation. Combined with demographic information, the speech markers could explain 11–14% of the variation of delusional ideation and schizotypy in Sample 1 and could discriminate between high-low schizotypy with high accuracy (0.72−0.70, AUC = 0.78–0.79) in Sample 2. The moderate to high retention rate, strong correlation of speech measures across manual and automated transcripts and sensitivity to psychotic-like experiences provides initial evidence that online collected speech in combination with automatic transcription is a feasible approach to increase accessibility and scalability of speech-based assessment of psychosis.

## Introduction

Psychotic illnesses, like bipolar disorder and schizophrenia, are a leading cause of disability with high associated societal costs and personal suffering [1]. Despite the considerable burden on society and healthcare systems, the causes of psychotic illnesses are still poorly understood, and treatment is often unsuccessful [2, 3]. Advances in digital technologies in a clinical setting, including the availability of remote, online, assessments, could improve various research and treatment-based outcomes. Examples include the ability to identify cognitive and behavioral factors that may relate to and predispose individuals toward experiencing psychosis symptoms. In turn, this could inform and improve treatment options. Specifically, as online and other remote assessments are more cost- and time-effective, a large number of individuals from a broad geographical area could be reached in a short period of time [4]. In addition, these assessments can be carried out in everyday familiar settings (e.g., at home), which avoids travel and the potential stigmatization of visiting a psychiatric clinic. In turn, this can make it easier to reach out to patients with a wide range of symptom profiles and monitor patients with severe symptoms [5].

A promising, cost-effective, and scalable marker of psychotic disorders, that can be assessed online, is speech disorganization. Speech disorganization can be assessed through automated methods rooted in Natural Language Processing (NLP)—an interdisciplinary subfield of linguistics, computer science, and artificial intelligence, enabling computers to process and analyze large amounts of natural language data. Semantic and structural measures of speech coherence are strongly associated with formal thought disorder, a key symptom of psychosis that denotes severe speech disorganization [6, 7]. Recent studies demonstrated that speech markers can be used to predict the development of psychosis, even in the nascent stages of illness, involving the ability to discriminate individuals at risk of psychosis who will develop psychosis from ones who will not at least six to twelve months before disorder onset [8–14]. As such, the detection of speech alterations might enable the identification of individuals who will develop psychotic disorders prior to clinical onset.

While this line of research offers a promising route toward improved identification and treatment of patients, former studies primarily use speech recordings collected in laboratory settings. As such, findings lack scalability, and may not be transferable to online assessment, where people have to self-record their speech. Also, most of these studies carried out dichotomous comparisons between small samples of completely healthy subjects and stereotypical patients, in whom the effects might be most apparent, but findings are not applicable for real-life conditions when people are presented with a wide range of symptom severity. It is, thus, unclear whether online-assessed speech using paradigms mirrored those used in laboratory-based psychosis research, renders data of sufficient quality, variance, and detail.

To facilitate the scalability of the aforementioned speech markers, it is important to use brief assessments, standardized prompts and to move beyond manual transcriptions, which are time-consuming and expensive, but remain the norm in psychosis research [9, 11, 15, 16]. Besides feasibility, current assessment procedures limit sample sizes in research which can lead to unproportionate overestimation of model performances given high-dimensional nature of language data. For example, the performance of speech-based machine learning models in psychiatry and neurology showed a negative correlation with the sample sizes of the study [17]. Utilizing standardized prompts and online assessment can mitigate the challenge of insufficient training and sample data in scientific studies and lead to more reliable, replicable, and realistic findings [17, 18].

In this study, we set out to test the feasibility of using short samples of online collected, self-recorded speech, in response to standardized prompts, in combination with automatic transcriptions. As a key step in establishing a procedure and evaluating whether it is fit for purpose, we assessed speech in relation to psychotic-like experiences, i.e., psychometric schizotypy and delusional ideation, in a large general population sample. The developmental connection between these traits and psychosis is supported by many genetic, neurobiological, cognitive, and behavioral similarities [19–22]. Results from longitudinal studies also suggest that measures of schizotypy and delusional ideation might be predictive of conversion to psychosis [21]. These traits are, therefore, likely associated with speech alterations similar to those observed in psychosis. In addition to feasibility, studying psychotic-like experiences provides valuable insights into the underlying neurobiological and psychological mechanisms that contribute to psychotic disorders. Investigating psychotic-like experiences in diverse populations allows for the identification of potential risk factors and protective factors, aiding in the early detection and prevention of psychotic disorders. By studying the general population, we can gain insights into a broader range of experiences, from mild and transient to those that might be indicative of underlying mental health conditions without the co-founding effect of hospitalization or medication.

Herein, we investigate the ability of a wide range of automatedly assessable speech markers, previously proposed in the literature that are extracted from online-collected speech to capture subclinical psychotic-like experiences.

Specifically, we hypothesized that:

1. Online speech recordings of eight minutes, and in response to automated prompts using participants’ own computer or smartphone, will render data of sufficient quality and quantity to extract semantic, syntactic and connectivity measures.
2. Speech markers extracted from automated vs manual transcriptions are highly related/correlated.
3. Subtle alterations in speech are associated with psychotic-like experiences i.e., psychometric schizotypy and delusional-ideation.

## Methods

### Participants and samples

All participants were recruited via the recruitment platform Prolific (https://prolific.ac/).

**Sample 1** consisted of data collected from the general population. Inclusion criteria included: aged 18–40 years, self-reported fluency in English. Participants were recruited using Prolific (https://prolific.ac/). People with any previous head-injury and/or diagnosed mental disorder were excluded from the study. While 723 participants started the experiment, 530 completed the study, resulting in a sample size of 530 participants before data quality checks.

To obtain the second sample (**Sample 2**), we first screened 1000 individuals for low/high scores on the 74-item Schizotypal Personality Questionnaire (SPQ) [23]. Following screening (please see below for details), participants from the sample with the top and bottom 100 scores (who also represent the upper and lower 10th percentile of the sample) were invited to complete the speech task. From these, 180 started the speech tasks.

The study received Ethical Approval through the King’s College London ethics committee (Reference: LRS/DP-20/21-22608).

#### Online speech data collection procedure

All speech data were collected using the online platform Gorilla (https://gorilla.sc/). Before the start of the speech task, informed consent was obtained, and participants were requested to test the microphone on the device used for the study. Eight images from the Thematic Apperception Test (TAT) depicting ambiguous scenes were presented for a duration of one minute each, during which participants were instructed to verbally describe the image. They were informed that their speech would be recorded and shared with researchers. Following 30 s, participants were presented with a second prompt, asking, “how does the image make you feel?”. This procedure was mirrored on the procedure used in several laboratory-based studies that used TAT images to elicit speech [7, 24] Importantly, picture description using TAT images was shown to be more sensitive to capture psychosis-relevant speech alterations compared to other speech elicitation methods, e.g., free speech [25].

#### Preprocessing and extraction of speech markers

Participants’ recorded speech samples were transcribed automatically using the online software Otter (https://otter.ai/). These end-to-end automated transcriptions were stored and untouched by humans and served as the “Automated transcription” samples for the study. Subsequently, these transcriptions were checked manually and corrected by eight independent transcribers, following a shared protocol about punctuation, signaling unrecognizable words, and excluding transcripts based on low quality. Also, a subset of speech samples was transcribed manually by the independent transcribers, without the involvement of Otter. These manually corrected transcriptions constituted the “Manual transcriptions” samples for the study. Having both types of transcription available enabled an assessment of the quality of transcription by the automated method. All speech markers (please see below) were extracted for both the automatic and manual transcripts.

### Speech connectivity

Following transcription, non-semantic speech graphs were generated using the SpeechGraphs software (http://www.neuro.ufrn.br/softwares/speechgraphs). Non-semantic speech graphs represent the sequence of words as nodes and the connection between words as edges. We can measure different parameters of these graphs to estimate speech connectivity. An important feature of the graphs is the recurrence pattern. Extracted connectivity features can determine markers of short-range recurrence (such as range recurrence e.g., loop of one node, loop of two nodes) and long-range recurrence (such as the largest connected component [LCC] and the largest strongly connected component [LSC]). Speech connectivity captures both semantic and syntactic information, measuring both the semantic coherence, syntactic complexity, grammatical complexity, and correctness of language use. Overall, connectivity measures provide a proxy for narrative planning [26]. We generated the following measures of speech connectivity: nodes, edges, repeated edges, parallel edges, the loop of one node, the loop of two nodes, the loop of three nodes, the largest connected component, the largest strongly connected component, average total degree, density, average shortest path, diameter, and average clustering coefficient according to the procedure developed by Mota and colleagues [12, 24]. This methodology involves controlling for verbosity differences by calculating the speech graphs measures using a sliding window (30-word window size with fifteen words overlap between windows; and averaging across all windows for each speech excerpt. The resulting connectivity measures will be compared to connectivity measures extracted from random speech obtained by performing one hundred random shuffles of words in each speech extract (see [14, 24]).

### Semantic coherence

Semantic coherence was measured by the approach described by Iter and colleagues [27]. After the removal of stop words and filler words (“um”), each word was represented as a vector, such that words used in similar contexts (e.g., “desk” and “table”) were represented by similar vectors. Vector representations were generated by using word embeddings from the pre-trained Google News model [28]. Smooth Inverse Frequency (SIF) methodology was then used to generate sentence embedding with a single vector for each sentence [27]. Finally, cosine similarities between adjacent sentences were calculated. Specifically, the coherence of each response was calculated as the mean of the cosine similarities between adjacent sentences. This approach also allowed us to explore additional semantic measures, including on-topic scores, tangentiality, and repetition [25]. These were calculated as the slope of the linear regression of the cosine similarities over time (ranging from −1 to 1) (tangentiality), the mean of the cosine similarities between each sentence and the a priori stimulus description (ranging from −1 to 1) (on topic) and as the maximum cosine similarity between all possible pairs of sentences (ranging from −1 to 1) (repetition) [25].

### Syntactic complexity

Syntactic measures were defined on the basis of Part Of Speech tagging (POS-Tag). This consists of labeling every word by its grammatical function where labels are attached to words according to a large corpus-trained classifier. For example, the sentence ‘The cat is under the table’ is tagged by the POS-Tag procedure as ((‘The’, ‘DT’), (‘cat’, ‘NN’), (‘is’, ‘VBZ’), (‘under’, ‘IN’), (‘the’, ‘DT’), (‘table’, ‘NN’)) where DT is the tag for determiners, NN for nouns, VBZ for verbs, and IN for prepositions. For every transcript, we calculated the POS-Tag information with the Natural Language Toolkit (NLTK) (https://www.nltk.org/) and assessed the frequencies of Comparative Adjectives, Possessive pronouns, WH-determiners, WH-pronouns, WH-adverbs and All tags, following the methodology of [29].

### Subclinical psychotic-like experiences

Subclinical psychotic-like experiences were measured using the [23] (SPQ), a well-validated psychometric tool to measure schizotypy in both people with and without psychosis, showing good reliability, validity, and long-term stability [30, 31] and the 21-item [32] (PDI), a psychometric assessment that captures three different dimensions of delusional ideation (distress, preoccupation, and conviction). In Sample 1, based on practical considerations, we used the brief, 17 items version of the SPQ [33], while in Study 2, we used the original, 74 items version [23].

### Statistical analyses

#### Automated and manual transcription comparison

To compare automated and manual transcriptions, we conducted correlational analyses on the mean of speech markers across the eight samples per participants extracted from manual and automated transcripts. Analyses were conducted on Samples 1 and 2 separately. Additionally, we measured the Word Error Rate (WER), Match Error Rate (MER), and Word Information Lost (WIL) for automatic compared to manual transcripts. WER is a common metric of the performance of a speech recognition or machine translation system, measuring the accuracy of the system capturing words correctly. MER is the proportion of word matches which are errors; in other words, it captures the probability of a given word match being incorrect. WIL is a simple approximation to the proportion of word information lost. These metrics range between 0–1, where lower values indicate better transcription quality.

### Relationship of speech markers with demographic measures, psychometric schizotypy and delusional ideation

We aimed to test the relationship of speech markers with demographic information as these factors are known to be strongly connected to, and underly psychosis-proneness and psychotic symptoms in general [34]. As some speech markers were not normally distributed, we conducted non-parametric correlation analyses between speech markers, age and education, and Mann–Whitney *U*-tests on speech markers and gender. For speech markers, we took the mean of the eight, one-minute-long speech files and grouped the data by participants.

To investigate the relationship between speech markers and psychotic-like experiences we conducted non-parametric correlation analyses between speech markers, schizotypy, and delusional ideation scores in Sample 1. We conducted a Mann–Whitney *U*-test on speech markers in Sample 2 to explore differences between low and high schizotypy groups. We applied the Benjamini–Hochberg procedure on the threshold of significance to correct for multiple comparison.

Considering the complexity of human language, we expected that on the psychosis spectrum, we would observe small changes across several language markers rather than more pronounced alterations in a few markers. To investigate the relationship between speech and psychotic-like experiences, we therefore conducted multiple regression analyses in Sample 1 and multiple logistic regression analyses in Sample 2 to predict schizotypy, delusional ideation scores (Sample 1) or low-high schizotypy group membership (Sample 2) from speech markers. In both samples, we first built a model with demographic variables as the only regressors, given the importance of these measures in terms of predictive power to explain the variance of psychosis-proneness [34]. In the second step, we added all speech markers extracted from the automatic transcriptions as predictors in the multiple regression to quantify additionally explained variance. In step three, we replaced the markers from the automated transcripts with the markers from the manual transcriptions. These steps allowed us to explore changes in model performance change between automatic and manual transcriptions.

## Results

### Sample characteristics

#### Feasibility

##### Sample 1

**Fig. 1:**
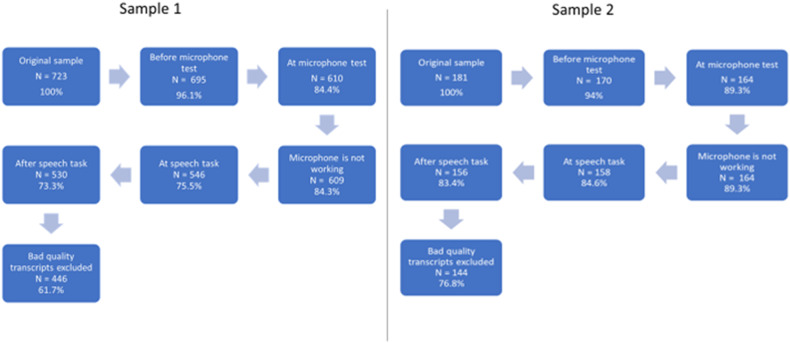
Testing procedure and drop-out rate of participants in Sample 1 and Sample 2.

[Table 1] Overall, 723 participants started the experiment, of which 530 completed it (26.7% dropout rate) (Fig. 1) (see details on drop-out ratio during the testing process in Supplementary material for details).

**Table 1:** Characteristics of samples.

Sample characteristics						
	Sample 1 (*N* = 446)		Sample 2 (*N* = 144)			
			High SPQ (*N* = 74)		Low SPQ (*N* = 70)	
	Mean	SD	Mean	SD	Mean	SD
**Age**	28.08	6.29	28.6	6.5	31.8	5.6
**SPQ total**	7.56	5.46	44.3	5.6	6.01	2.8
**PDI total**	3.83	3.00	5.4	3.1	1.6	2.0
	**Frequency**	**Percentage**	**Frequency**	**Percentage**	**Frequency**	**Percentage**
**Gender**						
*male*	153	34.30%	20	27.6%	19	27.8%
*female*	291	65.25%	54	72.4%	51	72.2%
*other*	2	0.45%	0	0%	0	0%
**Education**						
*Post-graduate university (finished)*	94	21.08%	12	15.8%	17	25%
*University (finished)*	159	35.65%	28	38.2%	37	52.8%
*University (ongoing)*	74	16.59%	12	15.8%	7	9.7%
*Professional training (finished)*	22	4.93%	0	0%	1	1.4%
*High school (finished)*	96	21.52%	19	26.3%	8	11.1%
*Not finished high school*	1	0.22%	3	3.9%	0	0%
**Type of device used for the experiment**						
*smartphone*	116	26.00%				
*computer*	326	73.09%				
*tablet*	4	0.89%

Transcripts of 84 participants (11.6% of the original sample; 15.85% of the participants who completed the experiment) were excluded from the analyses after the manual check of automated transcripts as they appeared to be of low quality (high level of noise, multiple speakers, not understandable speech) or people did not describe all eight TAT images or their other data on psychotic-like experiences were missing. The final dataset that was analyzed consisted of the data of 446 participants (see Table 1 in Supplementary material for details).

##### Sample 2

We screened the general population for schizotypal behaviors, using the previously validated Schizotypal Personality Questionnaire (SPQ) [23]. The total SPQ score was calculated as the sum of the 74 questions (yes = 1, no = 0) for each screened participant. 1000 participants were screened, of which 128 were excluded due to a failure of the attention check, not wanting to be re-contacted at follow-up, and/or indicating that they did not have a working microphone on their device, which would prevent them from completing the speech tasks in our study. Of this remaining sample (*N* = 872), the highest and lowest scoring 15% on the SPQ were re-contacted for the follow-up (*n* = 256). Of these, 181 started the speech experiment, of which *N* = 156 completed it (13.8% dropout rate) (see Details on drop-out ratio during the testing process in Supplementary material for details).

Transcripts of twelve participants (6.6% of the original sample) were excluded from analyses after the manual check of automated transcripts as they appeared to be of low quality (high level of noise, multiple speakers, not understandable speech). The final dataset for analyses comprised of 144 participants (See Table 1 in Supplementary material for details).

#### Comparison of automated versus manual transcriptions

##### Correlation of speech markers in Sample 1

For speech connectivity markers, the correlation coefficient between automated and manual transcriptions ranged between *r* = 0.549 and *r* = 0.838 (all *p* < 0.001). The largest Strongly Connected Component measure had the weakest correlation, while the Repeated edges marker had the strongest correlation between transcription methods (Fig. 1 in Supplementary material).

For semantic coherence markers, the correlation coefficient between automated and manual transcriptions ranged between *r* = 0.375, and *r* = 0.974 (all *p* < 0.001). The correlation coefficient was lowest for tangentiality and highest for the Number of words (Fig. 2 in Supplementary material).

For syntactic markers, the correlation coefficient between automated and manual transcriptions ranged between *r* = 0.808, and *r* = 0.973 (all *p* < 0.001). The lowest correlation coefficient was for frequency of Wh-determiners while the Number of all tags marker had the highest correlation coefficient (Fig. 3 in Supplementary material).

##### Correlation of speech markers in Sample 2

For speech connectivity markers, the correlation coefficient between automated and manual transcriptions ranged between *r* = 0.608 and *r* = 0.898 (all *p* < 0.001). The Largest Strongly Connected Component measure had the weakest correlation, while the Loop of one and Loop of three nodes measures had the strongest correlation between transcription methods (Fig. 4 in Supplementary material).

For semantic coherence measures, the correlation coefficients between automated and manual transcriptions ranged between *r* = 0.731 and *r* = 0.992 (all *p* < 0.001) in Sample 2. Tangentiality had the weakest correlation while the Number of words measured had the strongest correlation between transcription methods (Fig. 5 in Supplementary material).

For syntactic markers, the correlation coefficient between automated and manual transcriptions ranged between *r* = 0.923, and *r* = 0.992 (all *p* < 0.001). The frequency of personal pronouns marker had the lowest correlation coefficient while the Number of all tags marker had the highest coefficient (Fig. 6 in Supplementary material).

#### Word error ratio and transcription quality

##### Sample 1

In Sample 1, the Word Error Ratio was 0.209, the Match Error Rate was 0.199, and the Word Information Loss was 0.289 between automated and manual transcripts.

##### Sample 2

In Sample 2, the Word Error Ratio was 0.082, the Match Error Rate was 0.081, and the Word Information Loss was 0.115 between automated and manual transcripts.

### Relation of speech markers with demographic measures

In both samples, some speech measures showed a weak (<0.3) correlation with education and age.

In Sample 1, the Largest Strongly Connected Component (*t* = −2.429, *p* = 0.016, df = 432), and number of sentences were higher among females (*t* = −3.846, *p* < 0.001, df = 442) in automated transcriptions.

The number of words and all tags measures were significantly higher among females (*t* = −2.386, *p* = 0.017; df = 442; *t* = −2.491, *p* = 0.013, df = 444) (*t* = −2.437, *p* = 0.015; df = 444; *t* = −2.404, *p* = 0.017, df = 444) in both the automated and manual transcripts.

In Sample 2, the measure Loop of one node’s mean was higher among males, in both automated and manual transcripts (*t* = 4.936, *p* < 0.001, df = 142) (*t* = 4.315, *p* < 0.001, df = 142). Mean words per sentence was higher among males (*t* = 2.774, *p* = 0.006, df = 142) in automated transcripts.

See Supplementary material Tables 2–4 for the detailed results on the relation of speech markers and demographic variables.

### Relations of speech markers with schizotypy and delusional ideation

#### Sample 1

##### Speech connectivity measures

Parallel edges were significantly positively associated with delusional ideation for both the automated and the manual transcripts. Loop of one node was positively associated with delusional ideation in the automated transcriptions. Repeated edges, loop of two nodes, average total degree, density were significantly, positively associated, while nodes, largest connected component, diameter, and average shortest path were negatively associated with delusional ideation for the manual transcriptions, but not for the automated transcriptions (please see Table 2 for details).

**Table 2:** Spearman’s rho values of correlation between speech markers and schizotypy (SPQ) and delusional ideation (PDI) scores.

Correlation of speech markers with schizotypy and delusional ideation scores				
	Sample 1			
	SPQ		PDI	
	Manual	Automated	Manual	Automated
*Speech connectivity markers*				
Edges	0.075	0.046	0.55	0.053
Repeated edges	0.048	0.039	**0.123**^a^	0.085
Parallel edges	0.038	0.036	**0.147**^b^	**0.102**^a^
Loop of one node	0.079	0.081	0.059	**0.133**^b^
Loop of two node	−0.011	0.003	**0.098**^a^	0.075
Loop of three nodes	−0.003	0.020	0.082	0.008
Largest Connected Component	0.002	0.011	**−0,122**^a^	−0.036
Largest Strongly Connected Component	0.027	−0.029	0.077	−0.003
Average Total Degree	0.042	0.018	**0.161**^b^	0.073
Density	0.029	0.002	**0.148**^b^	0.046
Diameter	−0.003	0.045	**−0.118**^a^	−0.011
Average Shortest Path	0.008	0.046	**−0.108**^a^	−0.001
Average Clustering Coefficient	0.011	0.026	0.067	0.013
*Semantic coherence markers*				
Number of Words	0.028	0.030	0.018	0.030
Number of Sentences	0.065	0.070	0.066	0.064
Mean Number of Words per Sentences	−0.051	−0.051	−0.043	−0.041
Coherence	−0.032	0.002	−0.079	−0.031
Maximum Similarity	0.015	0.040	0.012	0.042
Tangentiality	0.023	−0.039	0.045	0.036
On Topic	**−0.130**^b^	**−0.171**^b^	**−0.135**^b^	**−0.162**^b^
*Syntactic markers*				
Frequency of all tags	0.010	0.027	0.003	0.017
Frequency of comparative adjectives	0.021	0.033	0.011	0.026
Frequency of personal pronouns	**−0.105**^a^	−0.079	**−0.130**^b^	**−0.110**^a^
Frequency of Wh-determiners	0.003	0.022	−0.044	0.005
Frequency of Wh-pronouns	−0.045	−0.048	−0.062	−0.036
Frequency of Wh-adverbs	−0.000	0.034	−0.003	0.059

^a^Correlation is significant at the 0.05 level (two-tailed) after applying the Benjamini–Hochberg procedure to correct for multiple comparison.

^b^Correlation is significant at the 0.01 level (two-tailed) after applying the Benjamini–Hochberg procedure to correct for multiple comparison.

Table displays Spearman’s rho values.

Significant associations are highlighted by bold fonts.

##### Semantic measures

On-topic score was significantly negatively correlated with schizotypy and delusional ideation in both the automated and in manual transcriptions. However, correlations between other semantic measures and schizotypy and delusional ideation were not significant for either the automated or the manual transcriptions. Please see Table 2 for all correlations.

##### Syntactic measures

Of the syntactic measures, only the frequency of personal pronouns was significantly negatively correlated with schizotypy. There was a negative correlation for both the automated and manual transcripts. There were no significant correlations between any of the syntactic measures and delusional ideation.

#### Sample 2

When we compared speech markers between high and low schizotypy groups, only the number of sentence markers (Semantic) showed a significant difference, and only in the automated transcripts Table 3. Other markers did not show significant differences between groups. Among manual transcripts, there were no significantly different speech markers.

**Table 3:** Differences in speech markers between high and low schizotypy groups in Sample 2.

Differences between high and low schizotypy groups in sample 2 Mann–Whitney *U*-test				
	Manual		Automated	
	W- statistics	*p*	W- statistics	*p*
*Speech connectivity markers*				
Nodes	2544.500	0.857	2543.000	0.853
Edges	2622.000	0.611	−	−
Repeated edges	2822.500	0.354	2750.500	0.522
Parallel edges	2731.500	0.573	2798.000	0.407
Loop of one node	2593.500	0.990	2434.000	0.533
Loop of two node	2415.000	0.485	2617.500	0.914
Loop of three nodes	2625.500	0.889	2742.000	0.545
Largest Connected Component	2542.500	0.851	2543.000	0.853
Largest Strongly Connected Component	2301.500	0.250	2329.500	0.299
Average Total Degree	2631.000	0.871	2640.000	0.843
Density	2594.000	0.989	2584.000	0.982
Diameter	2815.000	0.370	2638.500	0.848
Average Shortest Path	2727.000	0.585	2608.000	0.944
Average Clustering Coefficient	2593.000	0.992	2706.000	0.644
*Semantic coherence markers*				
Number of Words	2778.000	0.454	2585.000	0.986
Number of Sentences	3146.500	0.123	2618.000	0.022*
Mean Number of Words per Sentences	2492.000	0.697	2637.000	0.853
Coherence	2490.000	0.691	2605.000	0.954
Maximum Similarity	2760.000	0.498	2650.000	0.812
Tangentiality	2666.000	0.763	3034.000	0.065
On Topic	2228.000	0.148	2114.000	0.065
*Syntactic markers*				
Frequency of all tags	2594.500	0.987	2585.000	0.986
Frequency of comparative adjectives	2724.000	0.543	2734.000	0.517
Frequency of personal pronouns	2519.000	0.778	2514.000	0.763
Frequency of Wh-determiners	2387.000	0.417	2507.000	0.741
Frequency of Wh-pronouns	2312.500	0.268	2202.000	0.121
Frequency of Wh-adverbs	2220.000	0.140	2219.500	0.139

Mann–Whitney *U*-test.

*Difference is significant at the 0.05 level (two-tailed) after applying the Benjamini–Hochberg procedure to correct for multiple comparison.

Significant differences are highlighted by bold font.

### Linear regression

When we applied linear regression to predict schizotypy scores from Sample 1, a baseline model, including only demographic variables could explain 3% of the variance (*R*² = 0.033, Adjusted R² = 0.025, RMSE = 5.392, *F* = 4.098, *p* = 0.003) (Table 4). Including only speech markers as predictors, the model could explain 10 and 8% of the variance of schizotypy for automated and manual transcriptions, respectively (*R*² = 0.101, Adjusted *R*² = 0.036, RMSE = 5.273; *R*² = 0.076, Adjusted *R*² = 0.008, RMSE = 5.348) (Table 4).

**Table 4:** Changes in predictive value in multivariable linear regression, applied to predict schizotypy (SPQ) and delusionary ideation (PDI) scores in Sample 1.

Performance metrics					
	Only demographics	Only speech markers on automated transcriptions	Only speech markers on manual transcriptions	Demographics + speech markers on automated transcriptions	Demographics + speech markers on manual transcriptions
**SPQ**					
R^2^	0.033	0.101	0.076	0.132	0.111
Adjusted R^2^	0.025	0.036	0.008	0.059	0.036
RMSE	5.392	5.273	5.348	5.211	5.273
**PDI**					
R^2^	0.008	0.097	0.089	0.111	0.142
Adjusted R^2^	0.001	0.032	0.023	0.036	0.069
RMSE	33.465	31.97	32.108	31.912	22.4

After adding all semantic, connectivity, and syntactic speech markers from automated transcripts to the demographic model, performance has increased (*R*² = 0.132, Adjusted *R*² = 0.059, RMSE = 5.211, *F* = 1.799, *p* = 0.007), explaining 13% of the variance (Table 4).When using all semantic, connectivity, and syntactic speech markers from manual transcripts instead of automated ones, performance was slightly reduced (*R*² = 0.111, Adjusted *R*² = 0.036, RMSE = 5.273, *F* = 0.480, *p* = 0.050) as the model explained 11% of the variance (Table 4).

When we applied linear regression to predict delusional ideation, a baseline model, including only demographic variables could explain less than 1% of the variance, and the model did not make a significantly better prediction than a random one (*R*² = 0.008, Adjusted *R*² = 0.001, RMSE = 33.465, *F* = 0.939, *p* = 0.441) (Table 4). Including only speech markers as predictors, the model could explain 10 and 9% of the variance, for automated and manual transcriptions, respectively (*R*² = 0.097, Adjusted *R*² = 0.032, RMSE = 31.97; *R*² = 0.089, Adjusted *R*² = 0.023, RMSE = 32.108) (Table 4).

When we added speech markers from automated transcripts to the model, performance has significantly increased (*R*² = 0.111, Adjusted *R*² = 0.036, RMSE = 31.912, *F* = 1.476, *p* = 0.05), explaining 11% of the variance, performing significantly better than a random model (Table 4). When we used speech markers from manual transcripts instead of automated ones in the same model, performance increased (*R*² = 0.142, Adjusted *R*² = 0.069, RMSE = 2240.4, *F* = 1.958, *p* = 0.002) as the model explained 14% of the variance (Table 4).

### Logistic regression

When we applied logistic regression to predict low-high schizotypy group membership in Sample 2, a baseline model, including only demographic variables could classify 61% of cases correctly (Nagelkerke *R*² = 0.106, Tjur *R*² = 0.079, Cox & Snell *R*² = 0.079, AUC = 0.666) (Table 5). Including only speech markers as predictors, the model could classify 63 and 61% of cases correctly for automated and manual transcriptions, respectively (Nagelkerke *R*² = 0.183, Tjur *R*² = 0.135, Cox & Snell *R*² = 0.137, AUC = 0.703; Nagelkerke *R*² = 0.206, Tjur *R*² = 0.153, Cox & Snell *R*² = 0.154, AUC = 0.725).

**Table 5:** Changes in predictive value in multivariable logistic regression, applied to predict high-low schizotypy scores in Sample 2.

Performance metrics					
	Only demographics	Only speech markers on automated transcriptions	Only speech markers on manual transcriptions	Demographics + speech markers on automated transcriptions	Demographics + speech markers on manual transcriptions
Accuracy	0.611	0.625	0.611	0.701	0.722
AUC	0.666	0.703	0.725	0.778	0.788
Sensitivity/Recall	0.641	0.676	0.662	0.769	0.756
Specificity	0.576	0.571	0.557	0.621	0.682
Precision	0.641	0.625	0.613	0.706	0.738
F-measure	0.641	0.649	0.636	0.736	0.738
Nagelkerke R²	0.106	0.183	0.206	0.316	0.325
Tjur R²	0.079	0.135	0.153	0.236	0.248
Cox & Snell R²	0.079	0.137	0.154	0.236	0.243

When we added speech markers from automated transcripts to the demographic model, performance significantly increased (Nagelkerke *R*² = 0.316, Tjur *R*² = 0.236, Cox & Snell *R*² = 0.236, AUC = 0.778), classifying 70% of cases correctly (Table 5). When we used speech markers from manual transcripts instead of automated ones in the same model, performance increased (Nagelkerke *R*² = 0.325, Tjur *R*² = 0.248, Cox & Snell *R*² = 0.243, AUC = 0.788) as the model classified 72% of cases correctly (Table 5).

## Discussion

This study investigated the feasibility of assessing NLP-based speech markers through brief online tests in combination with automatic transcriptions to link the extracted speech markers to psychotic-like symptoms in the general population.

In the proposed procedure, dropout rates were smaller (13.8%) or equal compared to (26.7%) laboratory studies which used offline assessment or screening [35]. Participants appeared to be most sensitive to drop-out in the early stage of the experiment, before or during the microphone test. However, participants were unlikely to dropout because of the lack of working microphone or failed testing (1 or 0% of participants dropped out because of failed microphone test). Another part of the experiment in which a substantial number of people dropped out was the speech task itself which they (32% in Sample 1, 24% in Sample 2) did not complete. Finally, we had to exclude 12% (Sample 1) and 7% (Sample 2) of participants who completed the experiment because of poor quality transcripts, resulting from lack of speech content, too quiet recordings, or too high levels of noise. These findings might reflect on the motivation or technical skills of the participants (people who are not motivated, dropout early, or not completing the task that includes some extra technical effort) but might also reflect on the limitation of trust that people have in the assessment like giving access to their microphone or voice recordings.

Notably, the drop-out rate and bad-quality transcripts were much smaller in the second sample, which was acquired following pre-screening. It is, therefore, possible that dropouts in the first sample did not occur because of the recording component or the speech assessment, but rather reflected participants likely to dropout in any experiment. It must be noted though, that the second experimental protocol included more detailed instructions and guidance that were built in based on the experiences and feedback from participants in the first study. Therefore, it is also possible that giving more instruction and direct orders increases the feasibility of speech-based online assessments, resulting in fewer drop-outs during the study protocol and better-quality transcripts.

Word error ratio and automated transcription quality were acceptable in both samples which suggest the feasibility of this approach. Notably, the quality of transcriptions was higher in Sample 2. The differences in on-word error ratio and transcriptions quality between samples can be due to multiple reasons. A potential reason behind the different results is better data quality in Sample 2, as we provided more instructions in this data collection procedure to participants, which on its own might have resulted in better performance. Besides more instructions, another reason could be that the online data collection and experiment platform that we used (Gorilla.sc) updated its voice recording feature which might resulted in higher voice quality of the recordings. A third reason can be that participants in Sample 2 have been previously screened and reinvited to the speech-recording element of the study (see in Participants and Samples section) which can imply higher engagement with the study from them than from participants in Sample 1. Fourthly, data collection of Sample 2 happened around a 1 year later than the collection of Sample 1 and it is possible that on average, participants owned technologically more developed, higher-quality devices to record their voice.

Although more replication and qualitative exploration on the feasibility of speech-based online assessment would be needed, it seems that participants are already happy to and capable of engaging in the proposed online tasks and our results suggest that online, brief assessment of speech is feasible. This engagement and resulting data quality might be improved by increasing trust in the tasks and data usage, giving more direct guidance on completion and eliminating technological barriers from voice recordings. For further exploration and confirmation of current findings, qualitative studies with people with lived experience would be needed and beneficial to understand which elements of the proposed procedure caused dropout and why.

### Using automated speech transcriptions

To test whether the automatization of transcription is feasible in online, speech-based psychosis assessment, we tested the correlation of language markers (that are commonly used in psychosis research) between automatically generated and manually conducted transcripts.

Correlation coefficients ranged between 0.549 and 0.838 for speech connectivity markers, between 0.375 and 0.992 for semantic coherence markers and between 0.808, and r = 0.992 for syntactic markers. From the analyzed 25 markers, 19 had strong (r > 0.7) correlations between transcripts in both datasets, and 24 had strong correlations in the second dataset only, suggesting the feasibility of using automated transcription in psychosis-spectrum assessment and further research.

Notably, the correlation between markers were always higher in the second dataset, presumably because of better quality of the transcriptions (in Sample 1, all Word Error Ratio, Match Error Rate, and Word Information Loss were around 2–2.5 times higher than in Sample 2). Regarding trends in markers, measures that are sensitive to punctuation like Tangentiality, Mean of Words/Sentences, Coherence, or On Topic had the weakest correlations between automated and manual transcripts. These markers derive information on sentence-level [25], therefore using an automated transcription method that accurately detects sentence endings/beginnings is crucial for reliable use of these measures in combination with automatic transcriptions.

When comparing across the different categories of speech markers, the syntactic measures appeared to be the most replicable between automated and manual transcriptions whereas markers based on morgan semantics appeared to be more sensitive to errors in automated transcription. Speech connectivity markers that capture both semantic and syntactic information were moderately fallible for errors in automatic transcriptions with great variance between different markers. These results suggest that automated transcripts might preserve the underlying grammatical structure of the text while potentially containing significant errors capturing semantic information that is relevant to psychosis detection. Unfortunately, Automated Speech Recognition systems are optimized for general word error ratio [36]. Therefore, it is possible that while these systems get better at recognizing common words in everyday conversations in the general public, might not improve capturing the specific vocabulary that our task (describing ambiguous images) requires. It is also possible that although Automated Speech Recognition systems underperform in populations with poor mental health as these individuals often have modified paralinguistic profiles. Findings on the varying correlations of speech markers between automated and manual transcriptions can also imply that while some techniques and measures are useful and informative in manual transcriptions, the application of Automated Speech Recognition systems warrants the development of different speech features and the focus on different parameters of language use.

Also, our findings might suggest that in case of the implementation of automated transcription, former results of the psychosis and speech literature based on manually transcribed speech data would remain similar, if the measures involved in the study were based on syntactic analysis (e.g., [13, 14, 29]). However, former results might have significantly changed if the feature set were heavily derived from semantic information (e.g., [37–39]), which might question the scalability of these approaches. Therefore, we urge the replication of these studies with automated transcriptions of the recordings.

### The association between speech markers and psychotic-like experiences

On Topic score was negatively correlated with schizotypy and delusional ideation both in the automated and in the manual transcriptions, suggesting that people who have a high level of schizotypal traits or delusionary ideation might struggle to keep their speech focused and deviate from the initial topic often.

While other semantic markers did not appear to have a significant relationship with the outcome variables, many connectivity measures showed significant relationships with delusionary ideation. Connectivity features that imply short-term reoccurences in narrative (e.g., Loop of one, two, and three nodes) had a positive correlation with delusional ideation, while features that indicate long-term reoccurrence and referencing (e.g., average shortest path, diameter, largest connected component) had a negative relation with delusional ideation score, suggesting that people with delusional ideation tend to structure their narratives into smaller elements and might lack longer-term, broader discourse planning. From syntactic measures, the frequency of personal pronouns was negatively associated with outcome variables, while other measures did not show a significant relationship with outcome variables.

Notably, delusional ideation showed more significant relations to speech markers than schizotypy score—possibly because of the higher range of scores and less skewed distribution, but it is also possible that this trait correlates more strongly with speech abnormalities at this investigated early stage of the psychosis spectrum.

Surprisingly, in Sample 2, only the Number of sentence markers showed significant differences between groups. It can be because at this, subclinical stage of the psychosis spectrum, we expect small changes in speech between groups and our sample was not sufficiently powered to capture these changes. It is also possible, that at the subclinical stage, the changes in speech are too subtle to be captured by individual markers or might not be present yet. Interestingly, Number of sentences showed significant differences only in automated transcriptions. We assume that in that case, instead of conveying the information that they have been designed to capture, Number of sentences measure rather serve as a proxy of voice quality in the Automated Speech Recognition systems, that may correlate with symptoms.

When interpreting these findings, it is also important to consider that speech is a complex signal, and we are trying to identify subtle changes in a high number of markers than with large changes in a few ones.

For example, single speech measures alone might not be able to discriminate between groups at different levels of the psychosis spectrum or fallible for different demographic factors [40], while a complementary set of language measures can predict a wide variety of symptoms and functioning [41]. Therefore, the usability of speech-based assessment is likely more clearly reflected in the predictive power of the combination of different types of speech markers, especially if sample sizes are large enough to match the high-dimensional nature of speech-based modeling [17].

Comparing regression analysis in Sample 1 and Sample 2, in Sample 1 the model could explain 11–14% variation of outcome variables. Considering the limited range and skewness of both outcome and feature variables, also, as the lower quality of transcripts, it is possible that a model that is fitted on a higher ranged, more normally distributed feature sets, coming from a higher quality of transcripts can produce a better fit. At the same time, in Sample 2, the same speech markers could discriminate between groups with a moderate level of accuracy and sensitivity, especially if taking into account the remote and self-recorded nature of data collection and the fact, that we would expect subtle changes in speech in this early stage of the psychosis spectrum. It is also possible that more complex, non-linear classifying algorithms, used in machine learning could result in higher discriminatory power—however, the exploration of these falls out of the scope of the current, proof-of-concept study.

Comparing automated and manual transcriptions, variables that had a significant relationship with schizotypy and delusionary ideation slightly differed and there were more significant relationships in manual transcriptions. However, the direction and the magnitude of these relationships were similar. Also, in regression models, model performances did not differ much when we used automated transcriptions instead of manual ones. These findings suggest that automated transcription might be able to substitute manual transcripts, which could drastically scale up data collection and potential sample sizes in research.

Previous research applied to clinical samples, mostly focused on binary classification outcomes rather than continuous ones. These studies also used offline collected and longer speech samples with machine learning approaches but applied fewer speech markers which make it difficult to compare our results to these clinical studies. Nevertheless, studies that aim to discriminate healthy controls from individuals with schizophrenia spectrum or bipolar disorder based on speech markers of semantic coherence, connectivity, or syntactics reach an accuracy between 0.70 and 0.93 These results are usually higher than we achieved in our classification task in Sample 2 (0.701–0.722). However, these studies did not attempt to discriminate between more subtle changes than completely healthy speech and speech of people with a psychotic illness- which is a relatively easier but clinically possibly less informative classification task. Spencer et al. [14] used speech connectivity measures in logistic regression to discriminate between healthy, clinical high–risk and first episode of psychosis groups and achieved lower accuracy (0.50–0.57%), suggesting that the involvement or wider range of markers can help to detect speech abnormalities at earlier stages of psychotic disorders and stratify patients according to their risk.

### Limitations and further research

We assessed the feasibility of online, speech-based assessment of the psychosis continuum, for the first time using two relatively large, independent samples. We measured an extensive range of previously published speech markers, that have not been combined previously.

This study provides proof-of-concept for using online collected speech in psychosis research. The next step would be a similar, proof-of-concept study that evaluates the feasibility of online speech assessment in people at clinical high risk of psychosis, or those with a first episode of psychosis. This would make it possible to investigate whether subtle changes in speech might be predictive of psychosis-onset, which could eventually help contribute towards improved preventative care. Assessment of people with early psychosis would also allow for further investigation of the relationship between psychotic symptoms, socio-occupational functioning, and speech.

Notably, our study has been conducted on speech samples of fluent and native English speakers and the generalization of our findings and proposed procedure for other languages and cultural contexts could be challenging. Some of the investigated speech measures might be less or more connected to psychotic-like symptoms in different languages and the proposed data collection procedure might not as effective and feasible in non-American and non-European cultures. Further replications and investigations in different languages and cultural contexts would be needed before applying our procedure in a clinical or research context.

Although this study combined many text-based speech markers, there are some other measures that we did not include, such as metaphorical language use [37], poverty of content [13, 37], and referential cohesion [13, 37, 39]. Testing the usability of these measures in online settings with short speech samples would complement the work presented here. Furthermore, whilst our study focused on text-based language markers, numerous findings support the use of vocal, acoustic markers to assess psychotic symptoms from speech [10, 42–44], which were not included here. Other findings suggest the potential of other, remotely assessable markers coming from visual cues or passive sensing data of smart devices for monitoring or assessment [10, 42–47]. Testing these markers in online settings, especially in additive nature to text-based markers would be another important step to potentially improve the power and precision of automated, online assessment of the psychotic spectrum.

## Conclusion

This study provides proof-of-concept for using online collected speech in psychosis research. Our findings suggest online, automated assessment of speech and psychotic symptoms via speech is feasible. Specifically, using automated transcription and short, standardized prompt-based online assessment of speech appeared suitable to capture sufficient information for predicting subclinical symptoms. The usability of speech-based assessment seems to be more clearly reflected in the predictive power of the combination of speech markers rather than the usage of few, discriminatory speech markers.

Importantly, automated transcription can reliably replicate values of widely used speech markers compared to manual transcription. When comparing across the different categories of markers, the syntactic measures appeared to be the most replicable between automated and manual transcripts.

Future research should include proof-of-concept studies that evaluate the feasibility of online speech assessment in people with a psychotic disorder.

### Supplementary information

Supplementary material

## Data Availability

Participants’ data available for research purposes upon request in forms of transcriptions.
