# Supplementary material for: Towards a scalable approach to assess speech organization across the psychosis-spectrum -online assessment in conjunction with automated transcription and extraction of speech measures
Source: Transl Psychiatry. 2024 Mar 21;14:156. doi: 10.1038/s41398-024-02851-w (PMC10954690; doi:10.1038/s41398-024-02851-w)
Supplement: Supplementary file 1 — Supplementary material [file 41398_2024_2851_MOESM1_ESM.docx]

# **Supplementary material**

**Details on drop-out ratio during the testing process**

**Sample 1**

People who did not complete the experiment were most likely to drop out by not completing the microphone test (85 participants:44% of drop out, 11.7% of the original sample). Of these, 28 participants left the experiment before the start of the microphone test (14.5% of drop out, 3.9% of the original sample). There were two participants who recorded that their microphone did not work (1.04% of drop out, 0.1% of the original sample). Sixty-two participants left during the speech task (32.1% of drop out, 8.6% of the original sample) whereas another sixteen participants left after they had completed the speech task (8.3% of drop out, 2.2% of the original sample; Figure 1).

**Sample 2**

Participants who did not complete the experiment were most likely to drop out before the microphone test (11 participants (44% of drop out, 6% of the original sample). There were no participants who recorded that their microphone did not work but six participants exited the experiment during the microphone test (24% of drop out, 4.7% of the original sample). Six participants left during the speech task (24% of drop out, 4.7% of the original sample). Two participants left after they had completed the speech task (8% of drop out, 1.2% of the original sample) (Figure 1).

| **Feasibility markers** | | | | |
| --- | --- | --- | --- | --- |
|  | **Sample1** | | **Sample2** | |
| **N** | 723 | | 181 | |
| **Drop out** | 193 (26.7%) | | 25 (13.8%) | |
|  | **% of drop out** | **% of original sample** | **% of drop out** | **% of original sample** |
| **Before microphone test** | 14.5% | 3.9% | 44% | 6% |
| **At microphone test** | 44% | 11.7% | 24% | 4.7% |
| **Microphone does not work** | 1.04% | 0.1% | 0% | 0% |
| **At speech description task** | 32.1% | 8.6% | 24% | 4.7% |
| **After speech task** | 8.3% | 2.2% | 8% | 1.2% |
|  | **N** | **% of original sample** | **N** | **% of original sample** |
| **Excluded bad quality recordings** | 84 | 11.6% | 12 | 6.6% |

## **Table 1** Feasibility markers


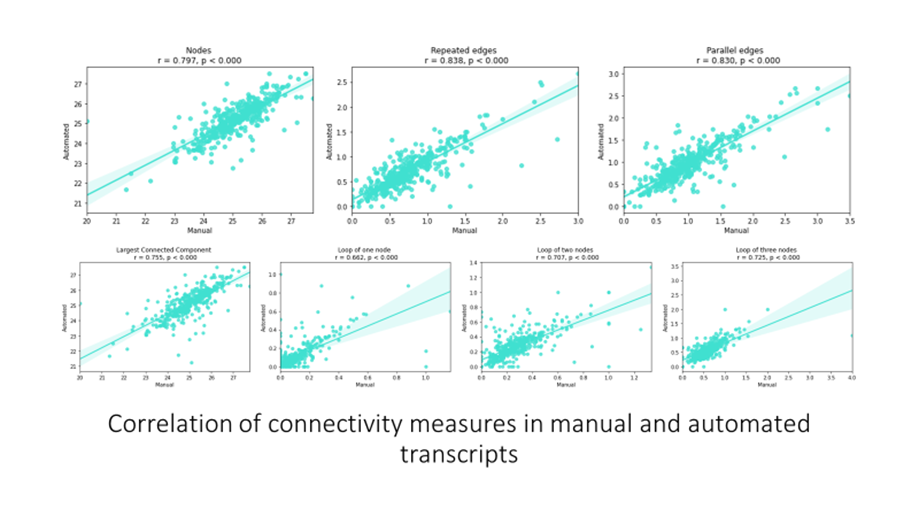


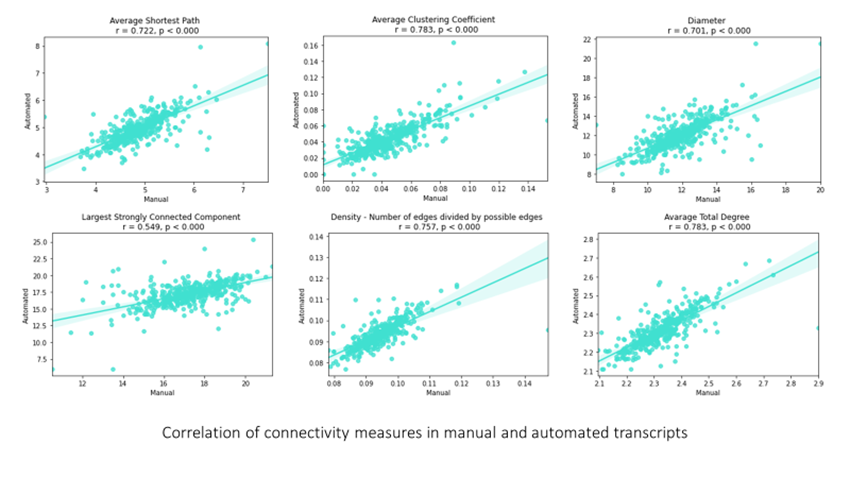


## **Figure 1** Correlations of speech connectivity markers in automated and manual transcriptions, Sample 1


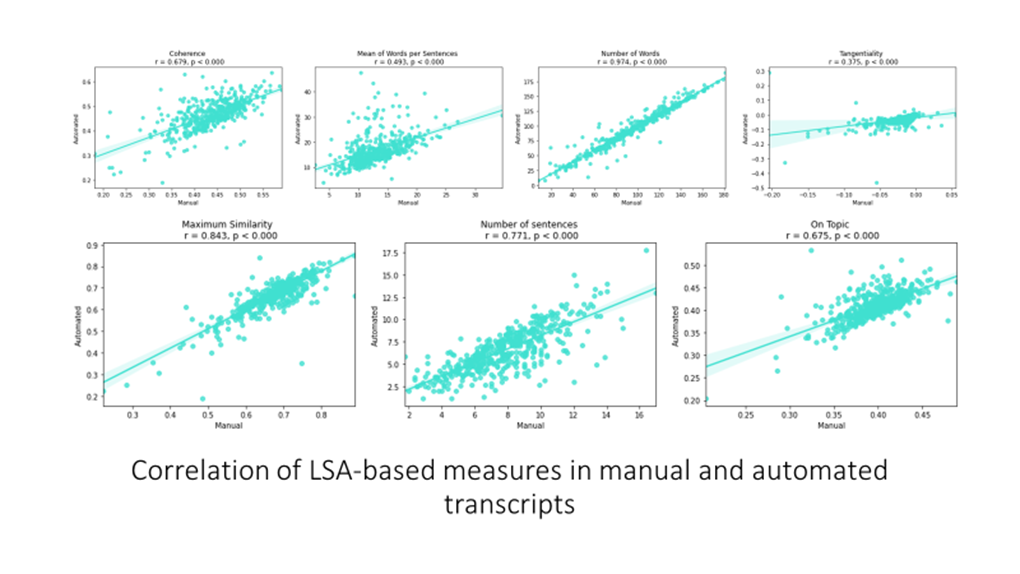


## **Figure 2** Correlations of semantic coherence markers in automated and manual transcriptions, Sample 1


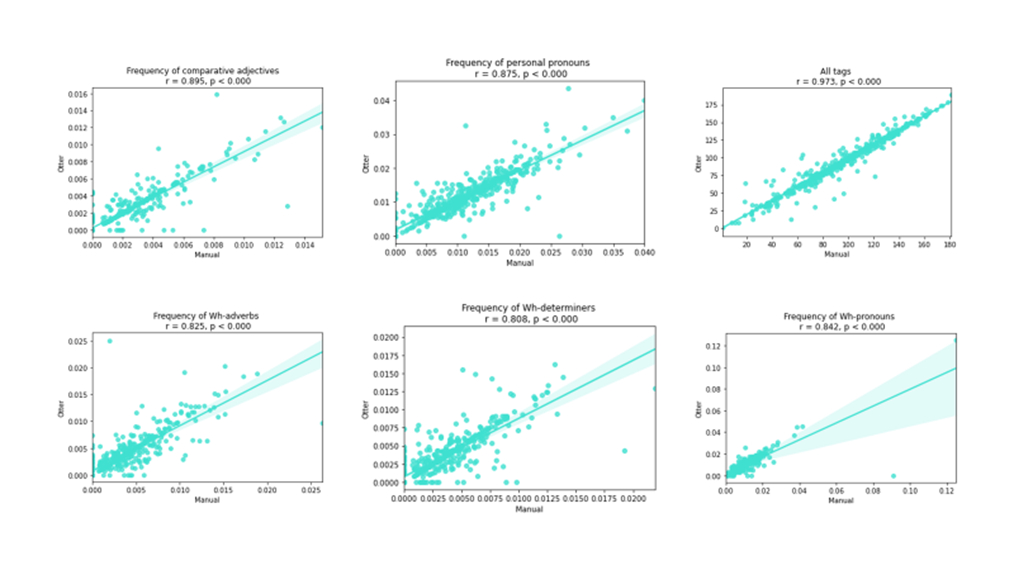


## **Figure 3** Correlations of syntactic markers in automated and manual transcriptions, Sample 1


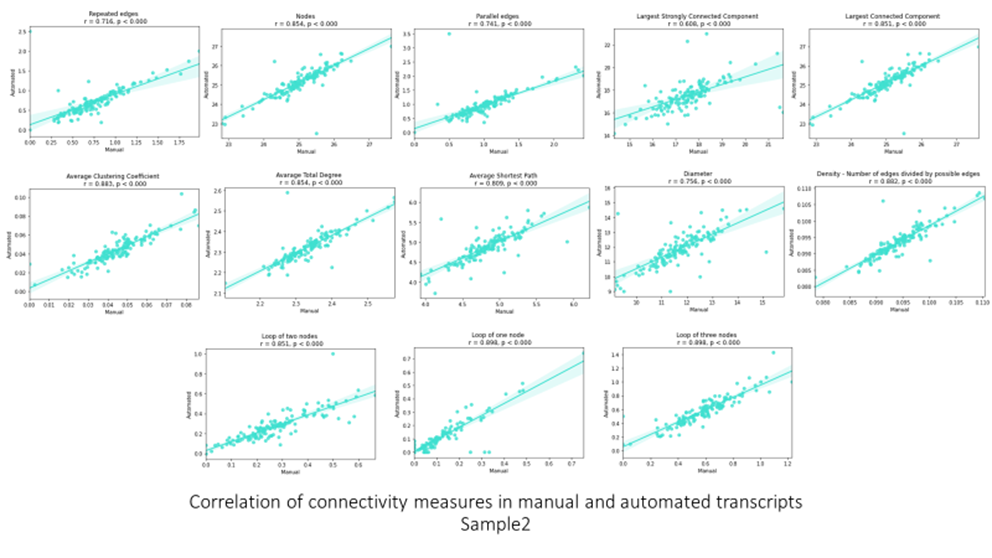


## **Figure 4** Correlations of speech connectivity markers in automated and manual transcriptions, Sample 2


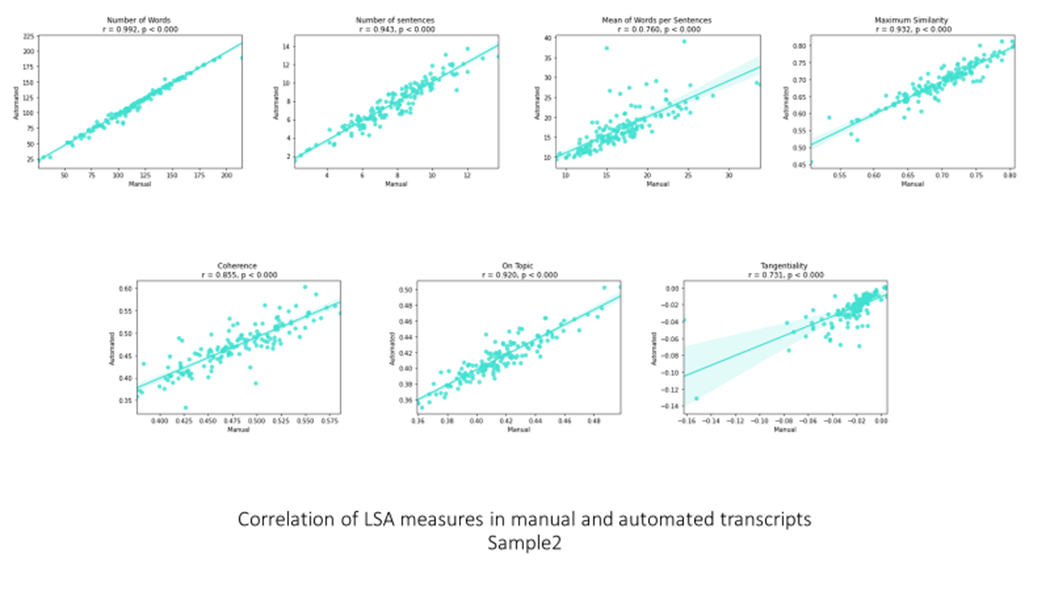


## **Figure 5** Correlations of semantic coherence markers in automated and manual transcriptions, Sample 2


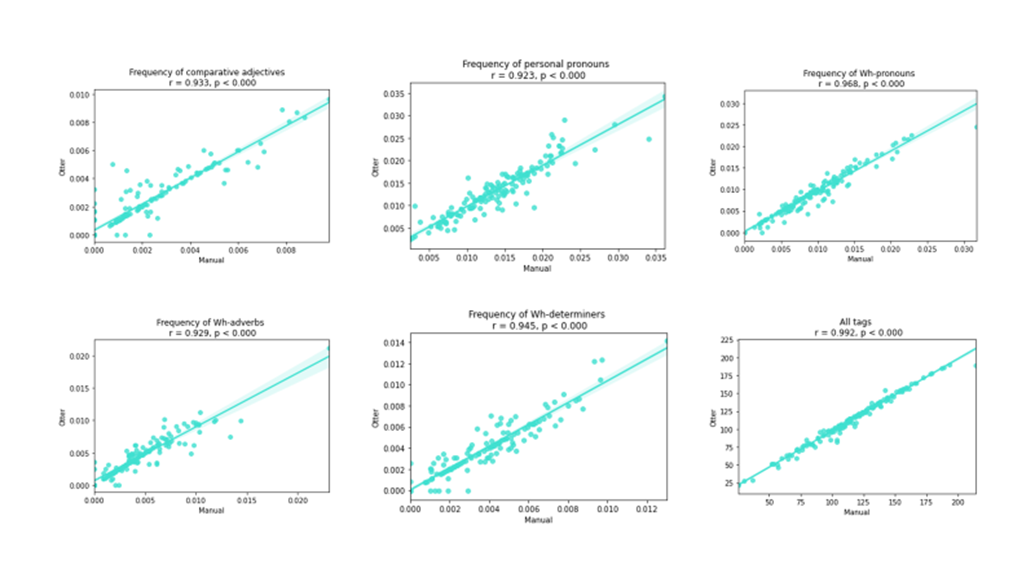


## **Figure 6** Correlations of syntactic markers in automated and manual transcriptions, Sample 2

## Relation of speech markers with demographic measures

### Age

| **Significant relationship between speech markers and age** | |
| --- | --- |
| **Sample 1** | |
| **Manual transcriptions** | **Automated transcripts** |
| Loop of one node (r = 0.113) | Frequency of Wh-adverbs (r = - 0.105) |
| Largest Strongly Connected Component  (r = 0.105) | Frequency of personal pronouns (r = 0.100) |
| Average Clustering Coefficient (r = 0.095) |  |
| Frequency of Wh-determiners measures  (r = -0.096) |  |
| **Sample 2** | |
| Nodes (r = 0.182) | Number of sentences (r = 0.213) |
| Largest Connected Component  (r = 0.180) | Mean of words per sentences (r = - 0.208) |
| Largest Strongly Connected Component  (r = - 0.201) | Coherence (r = - 0.183) |
| Average Total Degree (r = - 0.170) | Tangentiality (r = 0.222) |
| Density (r = - 0.170) | Frequency of Wh-pronouns (r = 0.244) |
| Diameter (r = 0.214) |  |
| Average Shortest Path (r = 0.208) |  |
| Frequency of Wh-determiners (r = -0.165) |  |
| Frequency of Wh-pronouns (r = 0.199) |  |

**Table 2**

**Significant relationship between speech markers and age**

### **Education**

| **Significant relationship between speech markers and education level** | |
| --- | --- |
| **Sample 1** | |
| **Manual transcriptions** | **Automated transcripts** |
| All tags ( r = 0.113) | All tags (r = 0.127) |
| Number of Words (r = 0.129) | Number of Words (r = 0.129) |
| Nodes (r = -0.118) | Nodes (r = - 0.137) |
| Largest Connected Component (r = - 0.116) | Largest Connected Component (r = - 0.147) |
| Average Total Degree (r = 0.119) | Average Total Degree (r = 0.120) |
| Density (r = 0.126) | Density (r = 0.122) |
| Diameter (r = - 0.149) | Diameter (r = - 0.135) |
| Average Shortest Path (r = - 0.158) | Average Shortest Path (r = - 0.141) |
| Mean Number of Words /Sentences  (r = 0.114) | Loop of two nodes (r = 0.113) |
| **Sample 2** | |
| Frequency of Wh-pronouns (r = - 0.193) | Frequency of Wh-pronouns (r = - 0.212) |

**Table 3**Significant relationship between speech markers and education level

### **Gender**

| **Significant differences in speech markers between genders** | |
| --- | --- |
| **Sample 1** | |
| **Manual transcriptions** | **Automated transcripts** |
| Number of words (t = -2.386) | Number of words (t = -2.491) |
| All tags (t = -2.437) | All tags (t = -2.404) |
|  | Largest Strongly Connected Component  (t = -2.429) |
|  | Number of Sentences (t = -3.846) |
| **Sample 2** | |
| Loop of one node (t = 4.936) | Loop of one node (t = 4.315) |
|  | Mean Words per Sentences ( t = 2.774) |

**Table 4**Significant differences in speech markers between genders

## Correlation of speech markers with each other


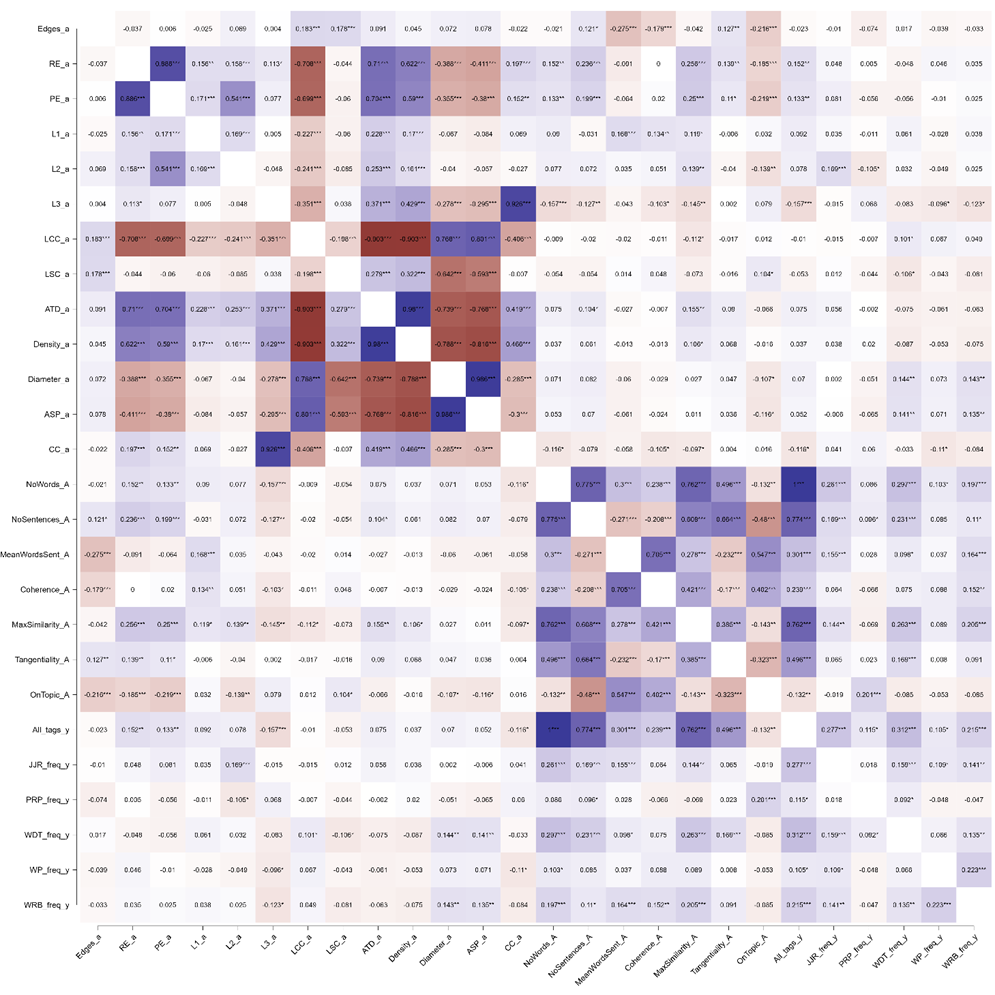


**Figure 7**

Correlation of speech features with each other in Sample 1

Spearman's rho heatmap


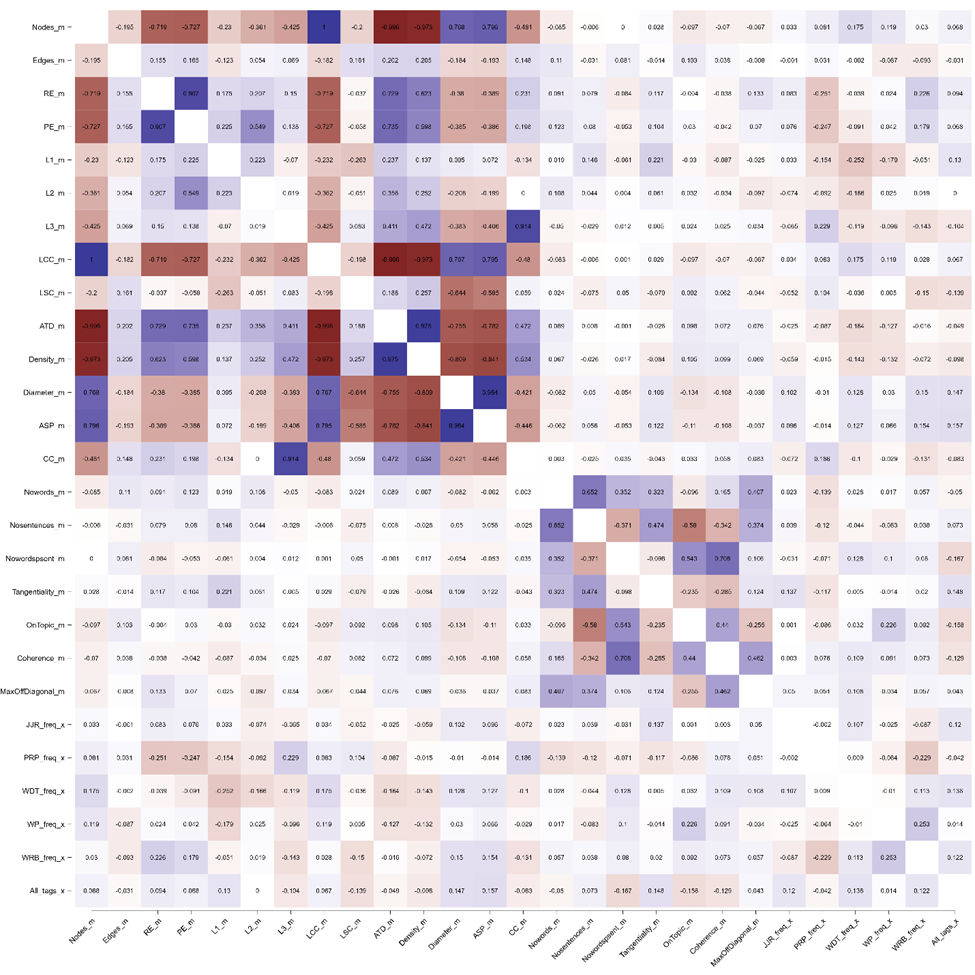


**Figure 8**

Correlation of speech features with each other in Sample 2

Spearman's rho heatmap
